# Mississippi Valley Medical Association

**Published:** 1896-09

**Authors:** Hanau W. Loeb

**Affiliations:** Secretary


### Mississippi Valley Medical Association.

The date of the meeting of the Mississippi Valley Medical Association has been changed to September 15, 16, 17, 18, in order to permit the members and their families to take the opportunity accorded by this change to make a pleasant tour through the Yellowstone Park, so justly celebrated as the wonderland of America.

Prominent resident members of the Association in St. Paul and Minneapolis are formulating plans for the special Yellowstone Park excursion trip, to leave on the evening of September 18th, arriving in Mammoth Hot Springs in the Yellowstone Park about noon on the following Sunday, and devoting the following five days to the wonders of this remakable region, returning to St. Paul Sunday, September 27th.

The cost of the trip, including all expenses west of St. Paul, will be announced in due season, but we are authorized to say that the figure will be a very favorable one, and we simply wish at this time to make the preliminary announcement of this most enjoyable feature of the St. Paul meeting, so as to give members the opportunity of making their plans in advance to join the party. It is desirable that there be a party of 100 or more, in order to obtain the benefit of the special train service in both directions.

It is urged that all members who desire to join the party should send their names to Dr. C. A. Wheaton, Chairman of the Committee of Arrangements, St. Paul, at as early a date as possible. If you desire to read a paper before the meeting, please send to me the title at once.

Very truly yours,

Hanau W. Loeb, Secretary.

